# Supplementary material for: Systems genetics uncover new loci containing functional gene candidates in Mycobacterium tuberculosis-infected Diversity Outbred mice
Source: bioRxiv. 2023 Dec 22:2023.12.21.572738. Preprint. [Version 1] doi: 10.1101/2023.12.21.572738 (PMC10769337; doi:10.1101/2023.12.21.572738)
Supplement: 1 [file NIHPP2023.12.21.572738v1-supplement-1.pdf]

bioRxiv preprint doi: https://doi.org/10.1101/2023.12.21.572738; this version posted December 22, 2023. The copyright holder for this preprint (which was not certified by peer review) is the author/funder, who has granted bioRxiv a license to display the preprint in perpetuity. It is made available under aCC-BY 4.0 International license.

Supplemental Figure 1. Mouse body weight following a low dose of aerosolized *M. tuberculosis* strain Erdman. Mice were infected with a low dose of *M. tuberculosis* strain Erdman by aerosol. Body weight of identically housed, age-, gender-, and generation-matched non-infected Diversity Outbred (DO) controls (n = 49) compared to baseline are shown over time (A). Body weight changes of Progressor DO mice (n = 195); Controller DO mice (n = 145); and C57BL/6J inbred founder strain mice that succumbed to pulmonary TB (n = 39), are shown over time compared to pre-infection baseline (B, C, D). All mice were weighed 1 to 3 days prior to *M. tuberculosis* infection, at least twice per week throughout infection, and immediately before euthanasia. Each line is the body weight expressed as a percentage of initial pre-infection body weight.

bioRxiv preprint doi: https://doi.org/10.1101/2023.12.21.572738; this version posted December 22, 2023. The copyright holder for this preprint (which was not certified by peer review) is the author/funder, who has granted bioRxiv a license to display the preprint in perpetuity. It is made available under aCC-BY 4.0 International license.

Supplemental Figure 2. Clinical correlates of survival due to pulmonary TB in Diversity Outbred (DO) mice following exposure to a low dose of aerosolized *M. tuberculosis* strain Erdman. Age-, gender-, and generation-matched DO mice were assigned to cages at random, and infected (or not infected) with a low dose of *M. tuberculosis* strain Erdman by aerosol exposure. All mice were initially weighed 1-3 days prior to infection, at least twice per week during infection, and immediately before euthanasia. Panel (A) shows retrospective analysis of initial body weights of Non-infected mice (n = 76) compared to pre-infection body weights of Progressors (n = 298) and pre-infection body weights of Controllers (n = 195), shown as box-and-whisker plots with the line at the mean for each group, and whiskers at the minimum and maximum. Data were analyzed by 1-way ANOVA with Tukey's multiple comparisons test ***p<0.001; ****p<0.0001. Panel B shows the rate of weight loss (gm/day) and duration of body weight (BW) loss in days were negatively correlated. Panel C shows the duration of BW loss was strongly, positively, and linearly correlated with survival by Spearman correlation analysis (r = 0.848 with dashed lines indicating the 95% confidence interval, 0.8204 to 0.8717, p<0.0001). Panel D is a correlation matrix to show how survival and 8 clinical indicators of pulmonary TB in Diversity Outbred mice correlate with each other. Only correlations with p-values <0.00001 are shown on the matrix. Cells marked by an "X" were not significantly correlated.

Supplemental Figure 3. Examples of necrotizing and non-necrotizing lesions in *M. tuberculosis* infected Diversity Outbred (DO) mice. Lung lobes were formalin-fixed, paraffin-embedded, sectioned, and stained with carbol fuschin and counterstained with hematoxylin & eosin. Panels A and B: High magnification images of necrotizing lung lesions. One example contains abundant pyknotic nuclear debris (A) and one example contains abundant fibrin, eosinophilic cellular debris, and less nuclear debris (B). Panels C and D: High magnification images of non-necrotizing lung lesions. Both examples contain predominantly viable cells, including macrophages, foamy macrophages, and foci of lymphocytes (400X).

Supplemental Figure 4. Common founder allele effects for four traits on distal chromosome 1 QTL. Founder allele effects of the four phenotypes with genetic mapping peaks having LOD > 6.0 on chromosome 1 at 155.36 Mb. All four phenotypes have similar allele effects. Each panel shows the founder allele effects for the phenotype listed in the title. Founders are on the horizontal axis and the standardized allele effect are on the horizontal axis.

Supplemental Figure 5. Receiver operator characteristic (ROC) curves for SVM training on traits used in gene prioritization. Each panel shows the true positive rate of the trained SVM as a function of the false positive rate for each trait. The area under the curve (AUC) is noted for each panel.

Supplemental Figure 6. Validation of QTL mapping results. Panel A: *M. tuberculosis* infected Diversity Outbred (DO) mice with one or more PWK/PhJ alleles at the mouse H-2 locus on chromosome 17 in *Dots8* (near 36 Mb) have significantly reduced survival compared to DO mice carrying other alleles (p = 0.00075, Cox-PH test). Kaplan-Meier curves of survival of *M. tuberculosis* infected mice carrying PWK/PhJ allele (red) or any other founder allele (black). Days of survival is shown on the horizontal axis and the proportion of mice surviving is shown on the vertical axis. Panel B: Lungs from *M. tuberculosis* infected CAST/EiJ inbred mice contain significantly more S100A8 protein (calgranulin A) than lungs of PWK/PhJ inbred mice. PWK/PhJ (red) and CAST/EiJ (green) inbred founder strains with 4-6 mice per strain time point, analyzed by Mann-Whitney *t*-tests within each time point, *p<0.05. Panel C: *M. tuberculosis* infected *S100a8* knockout (KO), heterozygotes (HET), wild-type (WT) C57BL/6 inbred mice were euthanized at the time points indicated on the X-axes, and *M. tuberculosis* lung burden assessed by CFUs with total combined 15-22 mice per genotype per time point from 2 independent experiments, shown as average and standard error of the mean. No significant (ns) differences were identified within each time point by mixed effects ANOVA with Tukey's post-test (p<0.05). Panel D shows *M. tuberculosis* burden in the lungs of PWK/PhJ (red) and CAST/EiJ (green) inbred founder strains with 4-6 mice per strain time point. No significant differences were identified by Mann-Whitney *t*-tests within each time point,

bioRxiv preprint doi: https://doi.org/10.1101/2023.12.21.572738; this version posted December 22, 2023. The copyright holder for this preprint (which was not certified by peer review) is the author/funder, who has granted bioRxiv a license to display the preprint in perpetuity. It is made available under aCC-BY 4.0 International license.

although there was a trend for higher bacterial burden at day 20 post infection in lungs from CAST/EiJ inbred mice as compared to PWK/PhJ.

Supplemental File 1 This file is an Excel workbook containing worksheets that list all protein-coding genes in each QTL with the functional candidates highlighted.

Supplemental File 2 This is an Excel fie that lists the top 10 functional candidates for each trait in each QTL.

Supplemental File 3 This is an Excel file that lists genes within chromosome 17 QTL.
